# Impact of acute sleep deprivation on laparoscopic performance: a prospective, randomized crossover trial

**DOI:** 10.1007/s00464-026-12872-9

**Published:** 2026-05-13

**Authors:** Jean-Paul Bereuter, Alexa Fries, Isabell Grummt, Mark Enrik Geissler, Rona Berit Geissler, Sofia Schmidt, Nathalie Buck, Juliane Weiß, Grit Krause-Jüttler, Jürgen Weitz, Marius Distler, Florian Oehme, Felix von Bechtolsheim

**Affiliations:** 1 Department of Visceral, Thoracic and Vascular Surgery, Faculty of Medicine and University Hospital Carl Gustav Carus, TUD Dresden University of Technology, Fetscherstraße 74, 01307 Dresden, Germany; 2 Surgical Skills Lab Dresden, Medical Faculty and University Hospital Carl Gustav Carus, TUD Dresden University of Technology, Fetscherstraße 74, 01307 Dresden, Germany; 3 Centre for Tactile Internet with Human-in-the-Loop (CeTI), TUD Dresden University of Technology, Dresden, Germany; 4 National Center for Tumor Diseases (NCT/UCC), Dresden, Germany

**Keywords:** Minimally invasive surgery, Sleep deprivation, Laparoscopic skill analysis

## Abstract

**Introduction:**

During night shifts, surgeons experience prolonged periods without sleep, which might impact their laparoscopic performance. Therefore, this study aimed to elucidate the effects of sleep deprivation on tissue handling and motion parameters during laparoscopic surgery.

**Methods:**

A total of 55 medical students and 20 surgeons participated in this single-center, prospective, randomized crossover trial. All students underwent standardized laparoscopic training until they reached proficiency. Afterward, all participants performed three different laparoscopic tasks twice, once sleep-deprived in the middle of the night and once well rested. Endpoints were tissue handling, defined by force exertion, instrument motion, task time, and error rate.

**Results:**

Sleep-deprived students and surgeons demonstrated a significantly lower mean force exertion but only in one task, respectively. Sleep-deprived students showed a significant slower speed of the non-dominant hand in all tasks. Surgeons were significantly slower with their dominant hand in all tasks and produced a significant shorter path length and smaller motion volume. In two of the tasks, the task completion time of sleep-deprived surgeons was significantly higher compared to the control. There was no difference regarding the occurrence of errors in both cohorts. In subgroup analysis comparing tired vs. less tired participants, both very tired students and surgeons showed a tendency, in part significant, toward higher force exertion.

**Conclusions:**

Prolonged periods of sleep deprivation appear to have an overall effect on laparoscopic skills regarding instrument motion. There is also an alteration of tissue-handling skills in terms of force exertion among very tired students and surgeons.

**Graphical Abstract:**

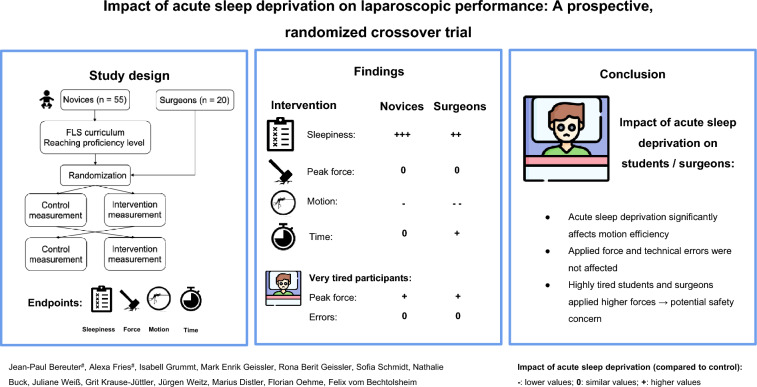

**Supplementary Information:**

The online version contains supplementary material available at 10.1007/s00464-026-12872-9.

Laparoscopic surgery is a widely utilized technique known for its advantages, including smaller incisions, reduced pain, and faster recovery, making it increasingly common, even in emergency situations [1, 2]. However, the demands for emergency operations, particularly during night shifts, introduce sleep deprivation as a critical stressor that can affect surgical performance [3–6]. This is particularly concerning, as laparoscopic procedures require high levels of manual dexterity, precise hand–eye coordination, and cognitive focus. Disruptions in these essential skills due to sleep deprivation can compromise both the safety and efficacy of these procedures, highlighting the need for further investigation into its impact on surgical outcomes [7–11].

A survey conducted by Anim et al. showed that 60% of surgeons worked more than 80 h per week [12]. These circumstances, especially in combination with shift work, not only affect sleep quality in the long run, but also affect surgeons’ health [13, 14]. In addition, another cross-sectional study by Trockel et al. reported that sleep deficiency was associated with self-reported clinically significant medical errors [15].

Sleep deprivation is defined as insufficient sleep and can be categorized based on sleep duration. Acute sleep deprivation refers to a temporary lack of sleep, often resulting from prolonged wakefulness such as during night shifts [16, 17]. In general, a lack of sleep causes physiological distress that deranges cardiovascular, neurocognitive, metabolic, and immunological processes [18]. This can lead to impairments in mood, cognitive and fine motor functions and a growing body of evidence suggests that these impairments can substantially affect surgical performance and therefore, potential patient outcome [3–6].

These findings raise concerns about the potential consequences of sleep deprivation on the cognitive and fine motor skills required to perform laparoscopic surgeries. Due to limited evidence concerning the effects of sleep impairment on laparoscopic surgical performance, this study investigated the impact of sleep deprivation on objective skill parameters, such as force exertion during tissue handling and instrument motion. To investigate the effects of sleep deprivation on laparoscopic performance under dry laboratory conditions, students were assessed in a controlled and standardized setting. To enhance clinical relevance and validate these findings, an additional cohort of surgeons was evaluated.

## Material and methods

This article was written in accordance with the CONSORT (Consolidated Standards of Reporting Trials) statement [19]. This study was conducted as a single-center, prospective, randomized crossover trial. Ethical approval was given by the ethics committee of Technische Universität Dresden (EK416092015), and written informed consent was obtained from all participants. No artificial intelligence (AI) tools were used in this trial in accordance with the TITAN Guidelines 2025 [20].

### Participants

This study was conducted at University Hospital Dresden between October 2023 and August 2024 and included a total of 55 medical students without previous experience in laparoscopic surgery and 20 surgeons. Students were selected to allow for a controlled and standardized study cohort, as they could be uniformly trained according to a modified Fundamentals of Laparoscopic Surgery (FLS) curriculum until reaching a predefined proficiency threshold [21]. In contrast, the inclusion of surgeons provided a clinically relevant cohort to validate the findings, as they may be less susceptible to external influencing factors and potentially more adapted to sleep deprivation due to professional experience. All participants had to perform three laparoscopic tasks in a standardized order (peg transfer, circle cutting, and surgical knot) twice, once with and once without sleep deprivation.

### Randomization

In this crossover trial, the order of intervention and control measurement was randomized. Participants were randomly assigned to either begin with the sleep-deprived measurement and then on a separate day under a well-rested (control) condition or vice versa.

### Sleep-deprived measurement (intervention)

We specifically aimed to investigate acute sleep deprivation, as this most accurately mirrors the conditions encountered by surgeons in a clinical night shift setting. To do so, the participating students were instructed to remain awake the night before the examination and to follow a standardized curriculum designed to simulate a night shift including physical and mental load. This included a normal workday with specific requirements: the students had to wake up by 8 a.m. and complete at least 4,000 steps by 6 p.m. This was followed by an exercise phase from 6 p.m. to midnight, during which they were required to complete an additional 2,000 steps and two written online surgery exams. A relaxation phase was provided before the intervention measurements. The student group was prohibited from sleeping before the intervention and from consuming coffee or any caffeine-containing products before the measurements. The participants were advised to avoid intensive physical exercise during the sleep-deprivation period. Therefore, the participants were contacted by the investigators several times during the night to guarantee coherence to the trial protocol.

In contrast, surgeons were tested during a combined day and night shift, starting at 10 a.m. and continuing until 08:45 the next morning. The surgeons were also advised to stay awake prior to measurement and to avoid intensive physical exercise. Here, the workload during the night shift was not influenced by the trial protocol.

The sleep-deprived measurements of both the student and surgeon groups were planned to be conducted between 1 and 2 a.m. However, the exact timing could vary slightly depending on the workload of the night shift or for logistical reasons. In addition, the measurement timing for sleep-deprived participants was intentionally selected to replicate conditions during night shift operation, aligning with findings from prior studies summarized by Reijmerink and colleagues [8].

### Control measurement

For control measurement, all participants were asked to follow their usual sleep routines prior to the scheduled examination. The control measurement was scheduled for the morning and at least 24 h after the last night shift ended. The participants were able to eat and drink as required for their personal liking.

### Measurement

The measurements were conducted in the surgical skills lab at University Hospital Dresden, providing a controlled environment free from external factors that could introduce bias. To mitigate learning effects or potential influences of prior sleep deprivation, an interval of at least 24 h was maintained between the two tests. Surgical performance in terms of force exertion and motion parameters was measured using a ForceSense device (Medishield B.V., Delft, The Netherlands) [6, 7]. The various tasks were mounted on a platform attached to a ForceSense device within a standard laparoscopic box trainer. This system allows for independent measurement of the motion of two laparoscopic instruments and records the force exerted (in Newtons) on task platforms.

### Assessment of sleep deprivation

To objectively assess the impact of sleep deprivation on the participants, vital parameters, such as blood pressure and heart rate, were measured before each test. Participants' sleep habits were further evaluated using standardized sleep-deprivation questionnaires, including the Pittsburgh Sleep Quality Index (PSQI), Epworth Sleepiness Scale (ESS), and Stanford Sleepiness Scale (SSS) [22–24]. The PSQI is a widely used self-report questionnaire that assesses overall sleep quality and disturbances over the past month. In addition, the ESS measures subjective daytime sleepiness by asking respondents to rate their likelihood of dozing off in various everyday situations, providing an index of general sleep propensity. In contrast, the SSS is a brief, momentary rating scale that captures an individual’s current level of alertness or sleepiness at a specific point in time. Together, these instruments offer complementary perspectives on acute and chronic sleep deprivation. Additionally, a simple, self-designed questionnaire was administered to capture details of sleep duration and perceived level of tiredness (no, moderately tired, and very tired).

### Primary endpoint—analysis of force parameters

Key force metrics included parameters such as peak force and mean non-zero force. The measured parameters were previously described by Hardon et al. and are defined as follows [25]:

- *Peak force [N]*: The maximal force exerted during a task.
- *Mean non-zero force [N]*: The average force excluding periods without any force exertion over the course of a task.

### Secondary endpoints

#### Analysis of motion parameters

Motion metrics included parameters such as mean instrument speed, path length and volume of motion. The motion parameters were measured separately for each hand using the ForceSense device and are defined as follows [25]:

##### Path length [mm]

Total distance traveled by the tip of the laparoscopic instruments during a task. To calculate path length, the euclidean distances between consecutive coordinates of the instrument tips were summed. The path length indirectly reflects the efficiency of movement.

##### Instrument speed [cm/s]

Average rate of motion of the laparoscopic instruments over the course of the conducted task. To measure the instrument speed, the three-dimensional position of each instrument tip was captured continuously throughout task performance. Instrument speed was then calculated as the first derivative of displacement over time and averaged across the task duration.

##### Volume of motion [mm^3^]

The spatial area traversed by the instruments mirroring the three-dimensional space covered by the instrument tip during a task. Volume of motion was computed by constructing a three-dimensional space enclosing all recorded instrument tip positions, thereby representing the total workspace utilized during the task.

#### Task completion time and surgical errors

The task completion time and number of surgical errors were recorded and provided a comprehensive basis for performance evaluation. Significant errors were defined for each task and recorded during each measurement based on previous studies [9–11]. Error occurrence across all tasks was recorded by a single blinded observer to ensure objectivity. An overview of the utilized error definition for each task is shown in Supplementary Table 1.

### Statistical analysis

Statistical analyses were performed using SPSS version 28 (IBM Corp., Armonk, NY, USA). The normality of continuous data was assessed using the Kolmogorov–Smirnov test and by inspecting the frequency distributions. Participant characteristics are presented as mean values with corresponding standard deviations (SD) for continuous variables or as frequency distributions for categorical variables. A formal a priori power calculation was not conducted for this study due to the absence of existing literature assessing the effect of sleep deprivation on surgical performance based on our endpoints. Appropriate statistical tests were applied based on data characteristics, including McNemar’s test and Wilcoxon rank test to compare conditions. No missing data were reported for the primary analysis, and a p-value of less than 0.05 was considered statistically significant.

## Results

### Basic participant characteristics

Fifty-five medical students and 20 surgeons participated in this single-center prospective randomized crossover trial. The basic participant characteristics are summarized in Table 1. Concerning the participants’ general amount of sleep, both students and surgeons demonstrated a similar amount of sleep on a regular basis (students: 7 h, surgeons: 6 h). Furthermore, participants' sleep quality (PSQI) and daytime sleepiness (Epworth Sleepiness Scale) were investigated. On average, both students and surgeons showed a similar sleep quality (PSQI: students: 5, surgeons: 6) and a normal level of daytime sleepiness (Epworth: students: 8, surgeons: 8). Based on the evaluated PSQI, 17 in 55 students (31%) showed a score above 5, which indicates a bad baseline sleep quality. In contrast, 11 in 20 surgeons (55%) were characterized by chronic sleep issues (Supplementary Table 2).

**Table 1:** Basic participant characteristics

Items	Students	Surgeons
Age [median (IQR)]	23 (22 – 27)	36 (32.25 – 39.75)
*Sex [n (%)]*		
Male	22 (40)	11 (55)
Female	33 (60)	9 (45)
*Academic year [n (%)]*		
3	9 (16.4)	–
4	38 (69)	–
5	6 (11)	–
6	2 (3.6)	–
*Years of surgical experience [n (%)]*		
1–6	–	9 (45)
7–12	–	6 (30)
13–18	–	5 (25)
Hours of standard sleep [median (IQR)]	7 (6 – 8)	6 (5 – 7)
PSQI [median (IQR)]	5 (4 – 6)	6 (4 – 7.75)
Epworth sleepiness scale [median (IQR)]	8 (6 – 12)	8 (4.5 – 12)

Numbers are shown as median (IQR) or n (%)

### Impact of sleep deprivation on participants

Under sleep-deprivation conditions, participants of the student cohort reported a significantly longer period of absence from sleep prior to the measurement than under control conditions (control: 5 h vs. sleep-deprived: 19 h, *p* < 0.001) (Table 2). Consequently, students had a significant higher score on the SSS (control: 2 vs. sleep-deprived: 4; *p* < 0.001) as well as higher levels of sleepiness (control: 0.47 vs. sleep-deprived: 1.47, *p* < 0.001) and tiredness (control: 0.55 vs. sleep-deprived: 1.33, *p* < 0.001) at the sleep-deprived measurement. Regarding the biophysiological parameters, blood pressure values did not differ between the measurement conditions, but the student group showed a significant lower heart frequency (control: 83 bpm vs. sleep-deprived: 75 bpm, *p* = 0.015) being sleep-deprived.

**Table 2:** Comparison of subjective sleepiness as well as objective vital parameters between the control and sleep-deprived measurement

		Control	Sleep-deprived	P-Value
Students	Duration sleep deprivation [hours]	5 (3—6.2)	19 (18.2—20)	** < 0.001**
	Stanford Sleepiness Scale	2 (1—3)	4 (3—5)	** < 0.001**
	Level of sleepiness [n (%)]			** < 0.001**
	Not	30 (54.6)	6 (10.9)	
	Moderately	24 (43.6)	17 (30.9)	
	Very	1 (1.8)	32 (58.2)	
	Level of exhaustion [n (%)]			** < 0.001**
	Not	28 (50.9)	7 (12.7)	
	Moderately	24 (43.6)	23 (41.8)	
	Very	3 (5.5)	25 (45.5)	
	Heart rate [bpm]	83 (71—90)	75 (65—87)	**0.015**
	Systolic RR [mmHg],	122 (116—130)	120 (111—131)	0.262
	Diastolic RR [mmHg]	75 (68—77)	72 (65—78)	0.911
Surgeons	Duration sleep deprivation [hours]	6 (4—8.1)	17 (17—20.1)	** < 0.001**
	Stanford Sleepiness Scale	2 (1—3)	3 (3—4)	**0.001**
	Level of sleepiness [n (%)]			** < 0.001**
	Not	10 (50)	0 (0)	
	Moderately	10 (50)	11 (55)	
	Very	0 (0)	9 (45)	
	Level of exhaustion [n (%)]			** < 0.001**
	Not	10 (50)	5 (25)	
	Moderately	9 (45)	9 (45)	
	Very	1 (5)	7 (35)	
	Heart rate [bpm]	67 (59—76)	72 (60—76)	0.493
	Systolic RR [mmHg]	120 (108—130)	115 (104—125)	**0.038**
	Diastolic RR [mmHg]	72 (66—82)	71 (68—83)	0.904

Significant p-values are highlighted in bold. Numbers are shown as median (IQR) or n (%)

Similar results were observed for the surgeon cohort (Table 2). Under sleep-deprivation conditions there was a significantly longer absence from sleep (surgeons: control: 6 h vs. sleep-deprived: 17 h, *p* < 0.001). Moreover, surgeons reported a significantly higher level of sleepiness (control: 0.5 vs. sleep-deprived: 1.45, *p* < 0.001) and tiredness (control: 0.55 vs. sleep-deprived: 1.15, *p* < 0.001) and higher SSS scores (control: 2 vs. sleep-deprived: 3, *p* = 0.001) during sleep deprivation. Concerning physiological parameters, no difference was observed in heart rate and diastolic blood pressure, but systolic blood pressure was significantly higher under control conditions (control: 120 mmHg vs. sleep-deprived: 115 mmHg, *p* = 0.038).

### Impact of sleep deprivation on force exertion

The peak force applied by students under sleep deprivation (Table 3) showed no significant differences compared to the control measurement in any of the tasks. Similarly, the students exerted a comparable mean non-zero force in the peg transfer and Circle cutting task, but showed a significantly lower force exertion during sleep deprivation in the Suture and Knot task (control: 0.84 N vs. sleep-deprived: 0.72 N, *p* < 0.001).

**Table 3:** Comparison of task completion time and force exertion between the control and sleep-deprived measurement for the cohort of students and surgeons

		Control	Sleep-deprived	*P*-Value
Students	*Peg transfer*			
	Peak force [N]	3.1 (2.4–3.9)	3.2 (2.2–4.3)	0.181
	Mean non-zero force [N]	0.82 (0.66–0.97)	0.84 (0.63–0.98)	0.556
	*Circle cutting*			
	Peak force [N]	2.9 (2.3–3.7)	2.6 (2.1–3.5)	0.212
	Mean non-zero force [N]	0.71 (0.57–0.98)	0.71 (0.59–0.93)	0.678
	*Suture and Knot*			
	Peak force [N]	3 (2.3–4.3)	2.7 (2.3–3.6)	0.156
	Mean non-zero force [N]	0.84 (0.68–0.94)	0.72 (0.66–0.81)	** < 0.001**
Surgeons	*Peg transfer*			
	Peak force [N]	2.4 (2.1–3.1)	2.3 (2.1–2.8)	0.185
	Mean non-zero force [N]	0.71 (0.61–0.84)	0.65 (0.58–0.75)	**0.042**
	*Circle cutting*			
	Peak force [N]	2.4 (2–4)	3 (2.3–3.6)	0.411
	Mean non-zero force [N]	0.75 (0.56–0.9)	0.76 (0.62–0.91)	0.765
	*Suture and Knot*			
	Peak force [N]	2.8 (2–4.6)	2.5 (1.9–3.9)	0.811
	Mean non-zero force [N]	0.76 (0.62–0.89)	0.7 (0.59–0.99)	0.184

Significant *p*-values are highlighted in bold

In the surgeons’ cohort, no significant difference in the applied peak force was observed between sleep deprivation and control in any task (Table 3). Concerning the mean non-zero force, surgeons applied significantly less force being sleep-deprived in the peg transfer task (control: 0.71 N vs. sleep-deprived: 0.65 N, *p* = 0.042) but not in the other tasks.

### Secondary endpoint—impact of sleep deprivation on motion skills

Regarding motion analysis (Table 4), sleep-deprived participants of the student cohort, showed a significantly lower mean instrument speed of the non-dominant hand in the peg transfer task (control: 3.4 cm/s vs. sleep-deprived: 3.1 cm/s, *p* < 0.001), the circle cutting task (control: 2.1 cm/s vs. sleep-deprived: 2 cm/s, *p* = 0.01), and the suture and knot task (control: 2.2 cm/s vs. sleep-deprived: 2.1 cm/s, *p* = 0.029). Mean speed of the dominant hand or motion volume of either hand did not differ in any task. In contrast, students showed significantly reduced path length in the circle cutting task under sleep deprivation (control: 962 cm vs. sleep-deprived: 881 cm, *p* = 0.027), while no significant changes were observed in the other tasks.

**Table 4:** Comparison of task completion time and laparoscopic motion performance between the control and sleep-deprived measurement

		Control	Sleep-deprived	*P*-Value
Students	*Peg transfer*			
	Task completion time [s]	133 (121—157)	137 (121—162)	0.725
	Mean speed non-dominant hand [cm/s]	3.4 (3–3.6)	3.1 (2.7–3.5)	** < 0.001**
	Mean speed dominant hand [cm/s]	3.9 (2.9–6.9)	3.4 (2.7–7.3)	0.246
	Total path length [cm]	969 (743–1352)	1370 (1214–1614)	0.725
	Volume of motion non-dominant hand [cm^3^]	7.1 (5.3–8.6)	6.4 (5.6–7.8)	0.159
	Volume of motion dominant hand [cm^3^]	21.7 (5.9–67.3)	8.6 (5.2–81.3)	0.183
	*Circle cutting*			
	Task completion time [s]	196 (154—236)	173 (147—227)	**0.048**
	Mean speed non-dominant hand [cm/s]	2.1 (1.8–2.6)	2 (1.8–2.4)	**0.01**
	Mean speed dominant hand [cm/s]	3.2 (2.4–5.5)	3.2 (2.3–5.1)	0.894
	Total path length [cm]	962 (723–1329)	881 (586–1260)	**0.027**
	Volume of motion non-dominant hand [cm^3^]	5.4 (3.9–7.2)	5.4 (3.3–6.7)	0.251
	Volume of motion dominant hand [cm^3^]	11.6 (8.1–67.6)	10.5 (6.6–57.7)	0.129
	*Suture and Knot*			
	Task completion time [s]	233 (161—346)	227 (179—337)	0.669
	Mean speed non-dominant hand [cm/s]	2.2 (1.9 – 2.5)	2.1 (1.9 – 2.3)	**0.029**
	Mean speed dominant hand [cm/s]	3.6 (2.7–6)	3.4 (2.7–5.2)	0.207
	Total path length [cm]	1467 (762–2232)	1273 (905–1878)	0.58
	Volume of motion non-dominant hand [cm^3^]	5.7 (4.8–9)	5.9 (4.5–8)	0.175
	Volume of motion dominant hand [cm^3^]	9.3 (6.8–56.7)	10.8 (6.9–58.7)	0.953
Surgeons	*Peg transfer*			
	Task completion time [s]	142 (122—159)	192 (144—223)	** < 0.001**
	Mean speed non-dominant hand [cm/s]	2.9 (2.8–3.4)	2.7 (2.4–3)	**0.009**
	Mean speed dominant hand [cm/s]	7.4 (6.4–9.3)	3.1 (2.5–6.39)	**0.003**
	Total path length [cm]	1458 (1217–1768)	1275 (733–1579)	0.1
	Volume of motion non-dominant hand [cm^3^]	6.6 (5.6–9)	6 (4.6–10.2)	0.852
	Volume of motion dominant hand [cm^3^]	81.1 (65.1–99.5)	10.3 (5.1–69.8)	**0.003**
	*Circle cutting*			
	Task completion time [s]	144 (115—180)	168 (117—235)	**0.015**
	Mean speed non-dominant hand [cm/s]	1.9 (1.7–2.4)	2 (1.8–2.3)	0.723
	Mean speed dominant hand [cm/s]	7.1 (5.1–7.7)	3 (2.3–4)	** < 0.001**
	Total path length [cm]	1073 (802–1319)	731 (543–1044)	**0.011**
	Volume of motion non-dominant hand [cm^3^]	4.9 (2.6–7.8)	5.5 (3.5–7.6)	0.062
	Volume of motion dominant hand [cm^3^]	77.8 (60.1–92.4)	10.3 (6.3–51.9)	** < 0.001**
	*Suture and Knot*			
	Task completion time [s]	265 (156—324)	241 (143—303)	0.744
	Mean speed non-dominant hand [cm/s]	2.1 (1.9–2.4)	2.1 (1.7–2.4)	0.266
	Mean speed dominant hand [cm/s]	5.2 (4–7)	2.8 (2.3–3.5)	** < 0.001**
	Total path length [cm]	1503 (1161–2597)	970 (668–1419)	**0.001**
	Volume of motion non-dominant hand [cm^3^]	6.1 (4.6–9.2)	7.7 (6.2–10.4)	**0.022**
	Volume of motion dominant hand [cm^3^]	57.6 (44.9–64.6)	7.3 (5.3–19.4)	** < 0.001**

Significant *p*-values are highlighted in bold

In the surgeon cohort, participants also showed a significant reduction of the mean speed of the non-dominant hand under sleep-deprivation conditions in the peg transfer task (control: 3.4 cm/s vs. sleep-deprived: 3.1 cm/s, *p* < 0.001), while no differences were observed in the circle cutting and the suture and knot tasks. Regarding the instrument speed of the dominant hand, surgeons showed a significant reduction under sleep-deprivation conditions in the peg transfer (control: 7.4 cm/s vs. sleep-deprived: 3.1 cm/s, *p* = 0.003), circle cutting (control: 7.1 cm/s vs. sleep-deprived: 3 cm/s, *p* < 0.001), and suture and knot (control: 5.2 cm/s vs. sleep-deprived: 2.8 cm/s, *p* < 0.001). In addition, surgeons exhibited significantly shorter total path lengths being sleep-deprived in the circle cutting (control: 1,073 cm vs. sleep-deprived: 731 cm, *p* = 0.011) and suture and knot tasks (control: 1,503 cm vs. sleep-deprived: 970 cm, *p* = 0.001). The volume of motion of the dominant hand reduced significantly under sleep deprivation in all tasks, whereas the volume of motion of the non-dominant was significantly higher under sleep deprivation in the suture and knot task (control: 6.1 cm^3^ vs. sleep-deprived: 7.7 cm^3^, *p* = 0.022).

### Secondary endpoint—impact of sleep deprivation on task completion time

With respect to task completion time (Table 4), participants of the student cohort were significantly faster under sleep-deprivation conditions in the circle cutting task (control: 196 s vs. sleep-deprived: 173 s, *p* = 0.048), while no differences were observed in the peg transfer and the suture and knot task.

On the contrary, the surgeons showed significantly higher task completion times under sleep deprivation in the peg transfer (control: 142 s vs. sleep-deprived: 192 s, *p* < 0.001) and the circle cutting task (control: 144 s vs. sleep-deprived: 168 s, *p* = 0.015) but not in the suture and knot task.

### Secondary endpoint—impact of sleep deprivation on surgical errors:

The analysis of error occurrence revealed no significant differences between the control and sleep-deprived measurements across all tasks in both the student and the surgeon cohorts (Supplementary Table 3).

#### Subgroup analysis: impact of sleepiness level

In a subgroup analysis of the sleep-deprived group, divided into less tired (non-tired & moderately tired) and highly tired participants (Table 5), it was found that especially highly tired students applied significantly higher forces in the Circle cutting task (less tired: 2.4 N vs highly tired: 2.9 N; *p* = 0.011) and the Suture and Knot task (less tired: 0.69 N vs. highly tired: 0.77 N; *p* = 0.012). Additionally, a trend toward higher force exertion could also be seen across all tasks. Apart from the mean instrument speed of the non-dominant hand in the peg transfer task (less tired: 2.9 cm/s vs. highly tired: 3.2 cm/s, *p* = 0.004), students showed no other altered instrument motion (Supplementary Table 4).In the surgeons’ cohort only a higher peak force in the peg transfer task (less tired: 2.2 N vs highly tired: 2.7 N; *p* = 0.044) could be observed among the highly tired participants. There were no differences between highly tired and non or moderately tired participants regarding errors in both students and surgeons (Supplementary Table 5).

**Table 5:** Subgroup analysis of force exertion of highly tired participants compared with moderate or non-tired participants among the group of students and surgeons

		Non / moderately tired	Highly tired	*p*-value
Students		(*n* = 23)	(*n* = 32)	
	*Peg transfer*			
	Peak force [N]	2.9 (1.8—4.3)	3.6 (2.6—4.8)	0.064
	Mean non-zero force [N]	0.73 (0.59—0.93)	0.9 (0.7—0.98)	0.062
	*Circle cutting*			
	Peak force [N]	2.4 (2—2.7)	2.9 (2.2—4.2)	**0.011**
	Mean non-zero force [N]	0.73 (0.59—0.93)	0.9 (0.7—0.98)	0.062
	*Suture and knot*			
	Peak force [N]	2.5 (2—3.5)	2.9 (2.5—3.6)	0.164
	Mean non-zero force [N]	0.69 (0.63—0.74)	0.77 (0.68—0.91)	**0.012**
Surgeons		(*n* = 11)	(*n* = 9)	
	*Peg transfer*			
	Peak force [N]	2.2 (1.8—2.3)	2.7 (2.1—2.9)	**0.044**
	Mean non-zero force [N]	0.66 (0.58—0.69)	0.64 (0.58—0.8)	0.518
	*Circle cutting*			
	Peak force [N]	2.5 (2—3.3)	3.2 (2.7—4.5)	0.11
	Mean non-zero force [N]	0.75 (0.59—0.94)	0.76 (0.71—0.92)	0.361
	*Suture and knot*			
	Peak force [N]	2.5 (1.8—3.5)	2.5 (1.8—5.9)	0.825
	Mean non-zero force [N]	0.69 (0.55—0.99)	0.7 (0.6—1.03)	0.658

Significant *p*-values are highlighted in bold. Numbers are shown as median (IQR)

## Discussion

Our study analyzed the impact of acute sleep deprivation on the surgical performance of students and professional surgeons as this most accurately mirrors the conditions encountered by surgeons in a clinical night shift setting. We observed that the induction of sleep deprivation was successful, as evidenced by the higher scores on the Stanford Sleepiness Scale and subjective self-evaluation in the sleep-deprivation group. Under sleep deprivation, no systematic differences in the applied maximum and mean non-zero forces were observed in most tasks. However, participants systematically demonstrated a reduced path length, lower motion volume, and decreased speed. Importantly, when further analyzing the sleep-deprived group by subdividing it into less tired and highly tired participants, we found that the highly tired individuals, especially students, applied more force.

While the changes in motion parameters could be at first glance interpreted as more economical movements, they may instead reflect psychomotor alterations and attentional lapses commonly associated with sleep loss. Consequently, the observed impact on instrument motion could be based on compensatory strategies which aim at minimizing errors despite reduced cognitive resources [26–29]. This adaptive response could also explain why error rates were not altered in our study. Participants may have unconsciously adjusted their movement patterns leading to more economical motions to increase the focus on precision. In contrast to our findings, previous studies reported increased task completion times that were caused by slower and inefficient instrument motions under sleep-deprived conditions [6, 30]. Although these studies did not examine laparoscopic performance, they also did not observe an effect on error rates. The participants of these studies may have been even more affected by sleep deprivation so that they showed a differing adaptive response in order to avoid error occurrence.

Importantly, highly fatigued participants, students more than surgeons, exerted excessive force with reduced fine motor control, producing movements that were rougher rather than more refined, a phenomenon also observed in other fatigue-related motor studies [31, 32]. This pattern is consistent with evidence that sleep deprivation impairs prefrontal cortex function and inhibitory control, leading to less regulated and less precise motor output [33, 34].

Although surgeons might generally be more accustomed to sleep deprivation due to their shift-based work schedules, they showed more alterations in surgical performance while being sleep-deprived in our study. A possible reason could be that sleep-deprivation induction protocols differed in our study. While students followed a structured and standardized protocol, surgeons were measured during a real night shift. However, a real night shift contains additional stress factors that could not be fully considered in the standardized simulation of sleep deprivation for the student group or because the medical cohort was significantly smaller, so that individual outliers could have influenced the results here. As a consequence, the group comparison between students and surgeons must also be interpreted with caution due to inconsistencies in the sleep-deprivation protocols. Findings from the surgeon cohort can be considered a validation of those observed in the student cohort. Although assessed under controlled dry-lab conditions, the results of surgeons might provide insight into the real-world effects of acute sleep deprivation on laparoscopic performance that could also be of clinical relevance.

## Clinical implications

A recently published review by Reijmerink et al., including 110 real-life studies and 24 simulator studies, showed that the negative effects of sleep deficiency on surgical outcomes were found in approximately 50% of simulator studies and one-third of real-life studies [3]. Simulator studies have found an impairment of cognitive functions and psychomotor skills under sleep-deprivation conditions [35, 36]. In addition, technical skill was decreased by up to 30% due to sleep deprivation in a controlled simulator environment [37].

Concerning the translation of these findings into clinically relevant settings, contradicting results can be found in the existing literature. A decent number of real-life studies showed an increase in complication rates as well as in-hospital mortality when patients have been operated by sleep-deprived surgeons [3, 38, 39]. However, the majority of real-life studies did not detect significant differences in patient outcomes between sleep-deprived and well-rested surgeons. For example, Ellmann et al. found that acute sleep deprivation in thoracic surgical residents did not significantly affect operative efficiency, morbidity, or mortality in cardiac surgery [40]. When negative effects were observed, they typically involved only a small subset of all the outcome measures assessed [3].

Importantly, many of the existing real-life studies lacked direct measures of sleep deprivation, such as validated questionnaires. In most studies, the time of day of surgery was used as a proxy for sleep deprivation [3]. In contrast, our study demonstrated that subjective sleepiness ratings were a sensitive indicator of altered tissue-handling skills. These findings suggest that incorporating validated sleep-deprivation assessments in future studies may help to detect clearer links between sleep deprivation and clinically relevant patient outcomes.

Based on these suggestions, future clinical studies should also address the question whether certain surgical procedures should be performed during night shifts or should be postponed until the next day. Our results indicate that surgeons with high subjective tiredness exhibit altered tissue-handling skills, suggesting potential risks to patient outcomes. It should therefore be investigated whether specific procedures demonstrate comparable or even better results when performed by rested surgeons the following day. As demonstrated for appendectomies, this issue warrants evaluation across a broader range of surgical procedures within randomized controlled trials [41].

## Limitations

A key strength of this trial was the inclusion of a substantial number of participants, comprising students (*n* = 55) with comparable levels of proficiency and surgeons (*n* = 20). However, the observed differences between these groups may have been influenced by the methodological setup of the study. While students followed a standardized sleep-deprivation schedule designed to simulate the stress of a surgeon’s night shift, this curriculum may not have fully replicated the complex origin of the stress experienced during actual night shifts. On the other hand, this experimental setup made it possible to study the effects of sleep deprivation in relative isolation from other potential sources of stress, at least in the subgroup of students. In contrast, surgeons were examined during their actual night shifts, which were not standardized, potentially introducing variability in the extent of sleep deprivation. It must be acknowledged that the relatively small sample size of surgeons, as well as the wide range of experience levels, may affect the stability of the results derived from this subcohort. While it is plausible that experienced surgeons cope better with sleep deprivation than junior residents, no significant differences were observed in our data. However, the inclusion of proficiency trained students combined with a standardized simulated night shift provided high-quality data, at least for this subcohort. Still, future studies should aim to include a more homogeneous group of participants to allow for clearer conclusions.

Moreover, this study focused on analyzing motor aspects of surgical performance, including motion and force parameters. Cognitive processes such as decision-making, attention and executive function were not assessed. Therefore, no conclusions can be drawn regarding their contribution to the observed effects. It is likely that sleep deprivation causes cognitive changes that may influence psychomotor performance, as demonstrated in a previous work [6]. Consequently, future studies should combine cognitive and motor assessments to further elucidate these effects.

For practical reasons, the assessment of sleep quality and quantity was deliberately kept as simple as possible in order to minimize disruption to the night shift, especially for the surgeon group. These simple, subjective tests are therefore also suitable for follow-up studies in a clinical setting, as they require relatively little effort and can be carried out independently. Nevertheless, there is a possibility that focusing on this type of assessment could lead to subjective bias of the results.

In addition, no formal multiple comparison correction such as a Bonferroni adjustment was applied. Although this approach is statistically justified for the primary endpoint, this correction could have been applied for the secondary outcome measures (path length, instrument speed, volume of motion) as they present inherently correlated measures of motor performance. However, due to the exploratory nature of the chosen secondary endpoints, no multiple comparison correction was conducted. Furthermore, a global multiple comparison correction would be overly conservative and increase the risk of Type II errors. Accordingly, unadjusted p-values are reported, and secondary and exploratory findings were interpreted with appropriate caution.

Despite these limitations, the experimental setup effectively induced states of acute sleepiness and fatigue, significantly impacting tissue-handling skills, particularly among the most tired participants in the subgroup of sleep-deprived participants. Moreover, this study uniquely analyzed the range of motion and force parameters to assess the effects of sleep deprivation on tissue-handling skills. Unlike previous studies, which primarily focused on task time and error rates, our investigation provided a more comprehensive evaluation of surgical performance under sleep-deprived conditions.

## Conclusion

Prolonged periods of acute sleep deprivation appear to show distinct effects on laparoscopic performance in trained students and surgeons. While only spotwise effects were observed concerning applied peak and mean non-zero force, motion parameters were consistently affected, with a lower instrument speed, path length and volume of motion. The observed effects were even more pronounced in the cohort of surgeons. Despite these alterations, error rates were not significantly different between the measurement conditions. Therefore, measurable alterations in motion efficiency were demonstrated but the clinical relevance is speculative. However, very high levels of sleepiness were associated with increased force application, indicating potential risks in severely fatigued surgeons. Overall, individual assessment of surgeon sleepiness may be warranted to mitigate fatigue-related alterations of surgical performance.

## Supplementary Information

Below is the link to the electronic supplementary material.

Supplementary file1 (DOCX 147 KB)
