# Supplementary material for: Impact of acute sleep deprivation on laparoscopic performance: a prospective, randomized crossover trial
Source: Surg Endosc. 2026 May 13;40(7):6005–16. doi: 10.1007/s00464-026-12872-9 (PMC13368893; doi:10.1007/s00464-026-12872-9)
Supplement: Supplementary file 1 — Supplementary file1 (DOCX 147 KB) [file 464_2026_12872_MOESM1_ESM.docx]

**Supplementary Material:**

**Supplementary Table 1:**

| **Task** | | **Error** | **Definition** |
| --- | --- | --- | --- |
| **Peg transfer** | | 0 | No triangle dropped |
|  |  | 1 | Triangle dropped |
| **Circle cutting** | | 0 | Cut deviates 0–5 mm outside the marked line |
|  |  | 1 | For each cut deviating >5–10 mm outside the marked line |
|  |  | 2 | For each cut deviating >10 mm outside the marked line |
| **Suture and knot** | |  |  |
|  | Accuracy | 0 | Needle passes through both target points |
|  |  | 1 | Needle passes through only one target point |
|  |  | 2 | Needle passes through neither target point |
|  | Adaptation | 0 | Both edges of the Penrose drain touch |
|  |  | 1 | Edges approximate but do not touch |
|  |  | 2 | No visible approximation of the edges |
|  | Tightness | 0 | Knot holds securely, even under manipulation |
|  |  | 1 | Knot becomes loose upon manipulation |
|  |  | 2 | Knot is visibly loose without manipulation |

**Supplementary Table 2:**

| **Items** | **Students**  **n (%)** | **Surgeons**  **n (%)** |
| --- | --- | --- |
| PSQI < 6 | 38 (69) | 9 (45) |
| PSQI ≥ 6 | 17 (31) | 11 (55) |
| Total | 55 (100) | 20 (100) |

**Supplementary Table 3:**

|  |  | **Control** | **Sleep deprived** | **p-value** |
| --- | --- | --- | --- | --- |
|  |  | **n (%)** | **n (%)** |  |
| **Students** | Peg transfer | 12 (21.8) | 18 (32.7) | 0.286 |
|  | Circle cutting | 18 (32.7) | 21 (38.2) | 0.607 |
|  | Imprecise stitches | 38 (69.1) | 36 (65.5) | 0.804 |
|  | Incomplete penrose adaption | 13 (23.6) | 8 (14.5) | 0.332 |
|  | Insufficient knot tightness | 1 (1.8) | 2 (3.6) | 1 |
| **Surgeons** | Peg transfer | 8 (40) | 8 (40) | 1 |
|  | Circle cutting | 7 (35) | 9 (45) | 0.687 |
|  | Imprecise stitches | 11 (55) | 14 (70) | 0.508 |
|  | Incomplete penrose adaption | 3 (15) | 5 (25) | 0.727 |
|  | Insufficient knot tightness | 0 (0) | 2 (10) | 0.5 |

**Supplementary Table 4:**

|  |  | **Non / moderately tired** | **Highly tired** | **p-value** |
| --- | --- | --- | --- | --- |
| **Students** |  | (n = 23) | (n = 32) |  |
|  | **Peg transfer** | |  |  |
|  | Mean speed non-dominant hand [cm/s] | 2.9 (2.6 - 3.1) | 3.2 (2.9 - 3.6) | **0.004** |
|  | Mean speed dominant hand [cm/s] | 2.9 (2.5 - 7.9) | 3.6 (2.7 - 7.1) | 0.423 |
|  | Total path length [cm] | 787 (665 - 1392) | 851 (727 - 1347) | 0.633 |
|  | Volume of motion non-dominant hand [cm^3^] | 5.5 (3.1 - 7.5) | 5.4 (3.5 - 6.5) | 0.891 |
|  | Volume of motion dominant hand [cm^3^] | 9.8 (6.6 - 64.1) | 11.3 (5.9 - 57.3) | 0.851 |
|  | Time [s] | 143 (131 - 172) | 131 (121 - 158) | 0.138 |
|  | **Circle cutting** | |  |  |
|  | Mean speed non-dominant hand [cm/s] | 1.9 (1.7 - 2.4) | 2.1 (1.8 - 2.5) | 0.437 |
|  | Mean speed dominant hand [cm/s] | 3 (2 - 5.1) | 3.4 (2.4 - 5.5) | 0.438 |
|  | Total path length [cm] | 881 (602 - 1,386) | 878 (571 - 1,189) | 0.798 |
|  | Volume of motion non-dominant hand [cm^3^] | 5.5 (3.1 - 7.5) | 5.4 (3.5 - 6.5) | 0.891 |
|  | Volume of motion dominant hand [cm^3^] | 9.8 (6.6 - 64.1) | 11.3 (5.9 - 57.3) | 0.851 |
|  | Time [s] | 189 (155 - 236) | 168 (132 - 224) | 0.246 |
|  | **Suture and knot** | |  |  |
|  | Mean speed non-dominant hand [cm/s] | 2.1 (1.9 - 2.3) | 2.2 (1.9 - 2.5) | 0.286 |
|  | Mean speed dominant hand [cm/s] | 3.2 (2.3 - 5) | 3.4 (2.7 - 5.3) | 0.609 |
|  | Total path length [cm] | 1,232 (864 - 1,869) | 1,383 (936 - 1,951) | 0.621 |
|  | Volume of motion non-dominant hand [cm^3^] | 5.8 (4.3 - 7.6) | 6.1 (4.9 - 8.5) | 0.366 |
|  | Volume of motion dominant hand [cm^3^] | 9.9 (7 - 71.4) | 11.9 (6.9 - 56.3) | 0.946 |
|  | Time [s] | 238 (185 - 337) | 224 (164 - 352) | 0.657 |
| **Surgeons** |  | (n = 11) | (n = 9) |  |
|  | **Peg transfer** | |  |  |
|  | Mean speed non-dominant hand [cm/s] | 2.6 (2.3 - 3) | 2.8 (2.7 - 3) | 0.196 |
|  | Mean speed dominant hand [cm/s] | 4.2 (2.4 - 8) | 2.7 (2.5 -3.4) | 0.271 |
|  | Total path length [cm] | 1360 (760 - 1814) | 888 (650 - 1495) | 0.305 |
|  | Volume of motion non-dominant hand [cm^3^] | 5.4 (4.5 - 8.7) | 7.7 (4.7 - 13.4) | 0.569 |
|  | Volume of motion dominant hand [cm^3^] | 41.5 (6.6 - 75.3) | 5.5 (4.1 - 23.3) | 0.102 |
|  | Time [s] | 192 (141 - 221) | 193 (138 - 254) | 0.849 |
|  | **Circle cutting** | |  |  |
|  | Mean speed non-dominant hand [cm/s] | 2.1 (1.6 - 2.4) | 1.9 (1.8 - 2.2) | 0.79 |
|  | Mean speed dominant hand [cm/s] | 3.1 (2.2 - 4.3) | 2.9 (2.4 - 3.7) | 0.732 |
|  | Total path length [cm] | 798 (582 - 1,087) | 637 (529 - 1,005) | 0.569 |
|  | Volume of motion non-dominant hand [cm^3^] | 7 (3.6 - 11.9) | 4 (3 - 7.3) | 0.119 |
|  | Volume of motion dominant hand [cm^3^] | 12.5 (7.5 - 75.5) | 7.5 (5.7 - 28.3) | 0.21 |
|  | Time [s] | 176 (117- 237) | 150 (117 - 238) | 0.97 |
|  | **Suture and knot** | |  |  |
|  | Mean speed non-dominant hand [cm/s] | 2.1 (1.8 - 2.5) | 2.1 (1.7 - 2.4) | 0.453 |
|  | Mean speed dominant hand [cm/s] | 2.8 (2.6 - 4.5) | 2.6 (2.1 - 3.3) | 0.233 |
|  | Total path length [cm] | 333 (281 - 532) | 434 (208 - 667) | 0.895 |
|  | Volume of motion non-dominant hand [cm^3^] | 9.1 (6.6 - 10.9) | 7.1 (5.8 - 9.5) | 0.233 |
|  | Volume of motion dominant hand [cm^3^] | 7.6 (5.8 - 25.8) | 5.6 (4.5 - 24.7) | 0.31 |
|  | Time [s] | 232 (140 - 275) | 252 (152 - 327) | 0.566 |

**Supplementary Table 5:**

|  |  | **Non / moderately tired** | **Highly tired** | **p-value** |
| --- | --- | --- | --- | --- |
| **Students** |  | (n = 23) | (n = 32) |  |
|  | Peg transfer | 8 (34.8) | 10 (31.2) | 0.351 |
|  | Circle cutting | 9 (39.1) | 12 (37.5) | 0.304 |
|  | Imprecise stitches | 12 (52.2) | 24 (75) | 0.206 |
|  | Incomplete penrose adaption | 2 (8.7) | 6 (18.8) | 0.501 |
|  | Insufficient knot tightness | 2 (8.7) | 0 (0) | 0.089 |
| **Surgeons** |  | (n = 11) | (n = 9) |  |
|  | Peg transfer | 5 (45.5) | 3 (33.3) | 0.349 |
|  | Circle cutting | 5 (45.5) | 4 (44.4) | 0.893 |
|  | Imprecise stitches | 9 (81.8) | 5 (55.5) | 0.438 |
|  | Incomplete penrose adaption | 3 (27.3) | 2 (22.2) | 0.329 |
|  | Insufficient knot tightness | 2 (18.2) | 0 (0) | 0.178 |
